# It Starts with a Question: A Strategic Communication Case Study to Expand Suicide Prevention Training Uptake in the United States

**DOI:** 10.1192/j.eurpsy.2026.12085

**Published:** 2026-07-31

**Authors:** S. A. Geegan, E. B. Hester, S. Guo, C. Rhein, D. Jaffe

**Affiliations:** 1 Integrated Strategic Communication; 2 Graduate Program in Communication, University of Kentucky, Lexington, United States

## Abstract

**Introduction:**

In Kentucky, a US state with high suicide rates, an academic medical center launched a statewide initiative to improve access to *Question, Persuade, Refer* (QPR). Widespread adoption of suicide prevention trainings like QPR remains a challenge. This case explores how strategic communication was useded to bridge the awareness–action gap and scale QPR uptake at the community level.

**Objectives:**

This case study evaluates the effectiveness of a communication campaign aimed at increasing online QPR uptake among adults in a high-risk, largely rural region of the US. Aims included assessing campaign reach, training completion, and the role of media integration and message framing.

**Methods:**

The campaign was informed by two theoretical frameworks: cognitive dissonance and the PESO (paid, earned, shared, owned) media model. Cognitive dissonance theory posits that individuals are motivated to resolve psychological unease when they perceive inconsistencies between their values and behaviors. Campaign messaging intentionally evoked dissonance by highlighting tensions between the value people place on protecting loved ones and their lack of preparedness to intervene in a mental health crisis (see example post: Figure 1).

The PESO model structured campaign execution:

- **Paid:** digital advertisements on Google and Meta
- **Earned:** op-eds and news coverage in state/regional outlets
- **Shared:** community partnerships and third-party amplification
- **Owned:** institutional websites, newsletters, and podcasts.

A mixed-methods approach was used to evaluate campaign performance from Sept. 2024 to March 2025. Quantitative data on media reach, impressions, click-throughs, and training completions were collected through analytics tracking and training platform logs. Qualitative data, including field notes from community events and presentations, were analyzed thematically to understand audience reactions and barriers to engagement.

**Results:**

Over the 6-month campaign period, **1,045 individuals completed the training**, marking a **181% increase** from the six months prior to the campaign (n = 372). Community participation rose from **112 to 445**—a **297% increase**. Internal stakeholder completions grew from 260 to 600. The campaign’s **cross-platform communication strategy** (summarized in Table 1) met or exceeded all objectives. Notably, surges in QPR uptake aligned with periods of heightened earned media.

**Image 1:**
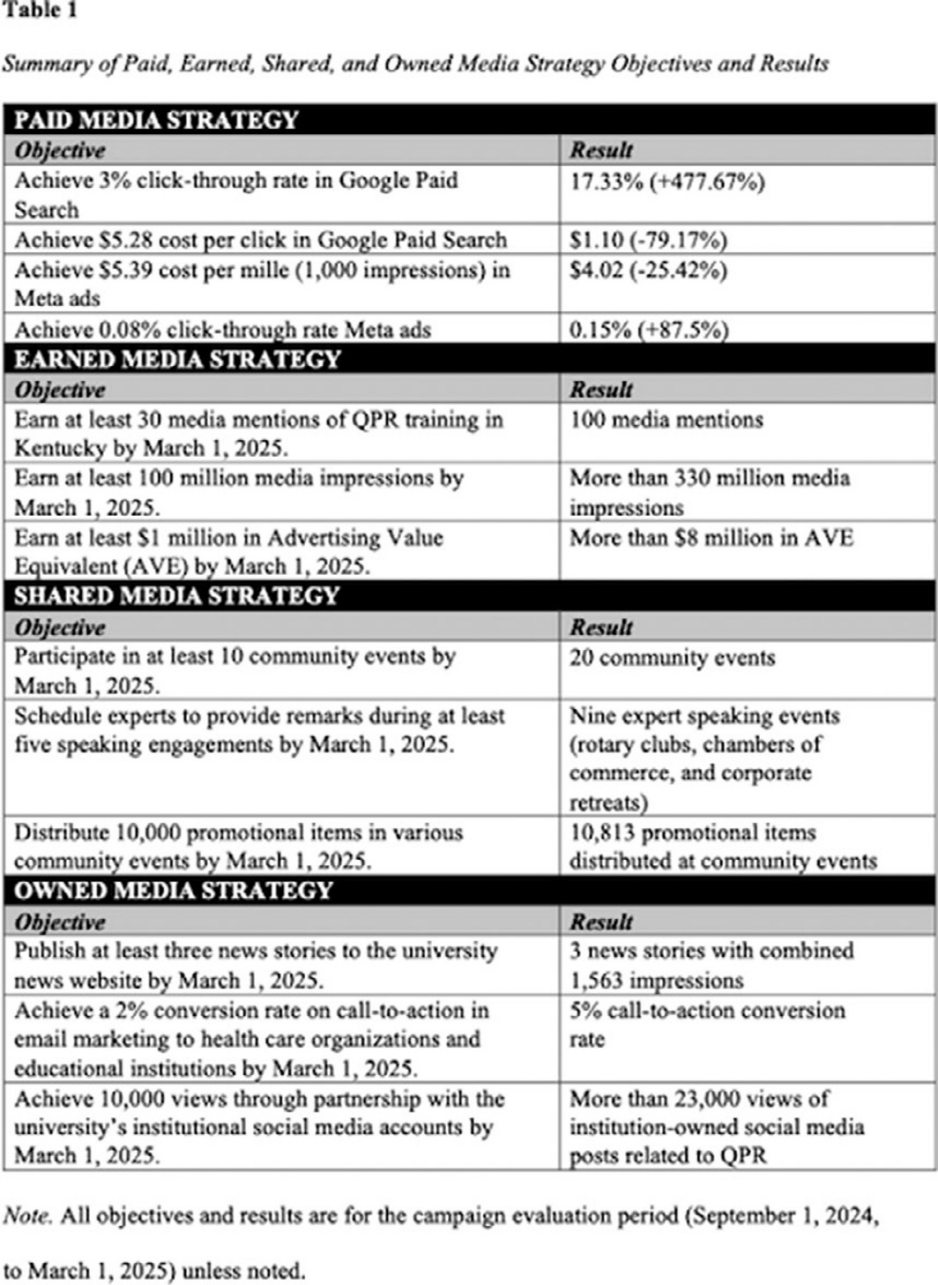
Image 1: Long description.

**Image 2:**
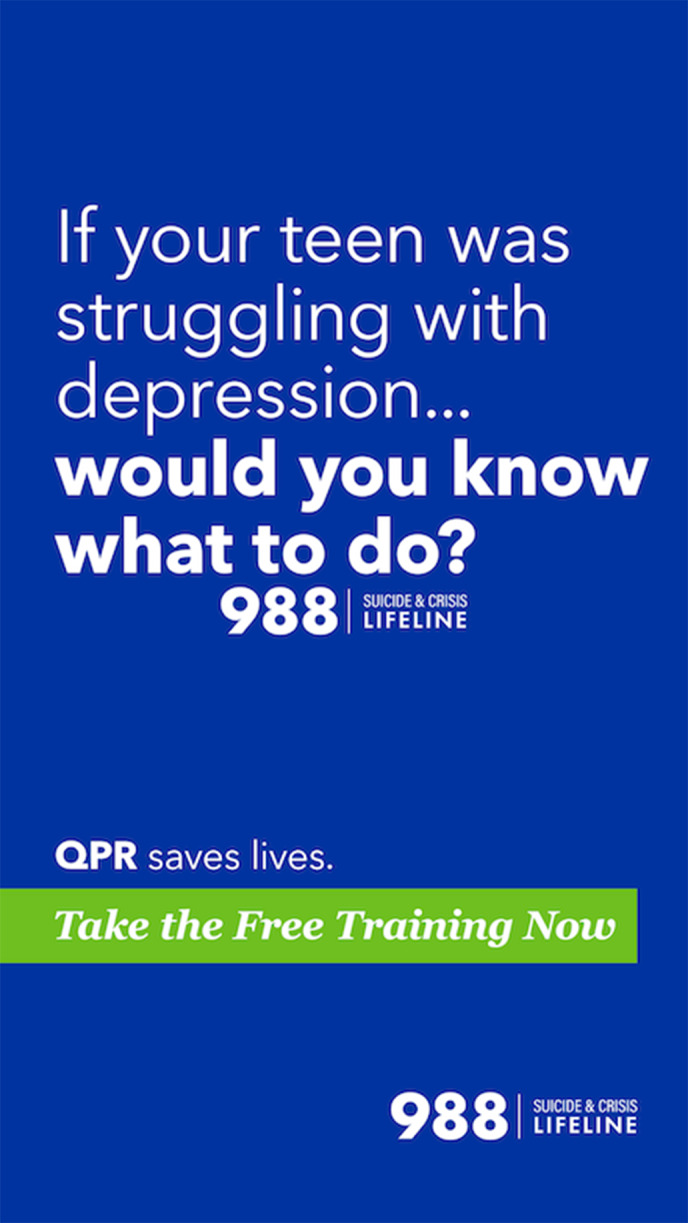

**Conclusions:**

This case illustrates how theory-driven, multi-channel communication can expand access to suicide prevention training and reframe it as a shared public responsibility. By integrating dissonance-based messaging with a PESO media strategy, the campaign effectively mobilized community engagement and reduced stigma. The model offers a scalable, transferable approach for addressing suicide risk in diverse settings, with implications for both practice and future research on communication strategies in mental health.

**Disclosure of Interest:**

None Declared

## Long descriptions

### Image 1: Long description

The table is divided into four sections.

1. PAID MEDIA STRATEGY

* Objective: Achieve 3% click-through rate in Google Paid Search. Result: 17.33% (+477.67%).

* Objective: Achieve $5.28 cost per click in Google Paid Search. Result: $1.10 (-79.17%).

* Objective: Achieve $5.39 cost per mille (1,000 impressions) in Meta ads. Result: $4.02 (-25.42%).

* Objective: Achieve 0.08% click-through rate Meta ads. Result: 0.15% (+87.5%).

2. EARNED MEDIA STRATEGY

* Objective: Earn at least 30 media mentions of Q P R training in Kentucky by March 1, 2025. Result: 100 media mentions.

* Objective: Earn at least 100 million media impressions by March 1, 2025. Result: More than 330 million media impressions.

* Objective: Earn at least $1 million in Advertising Value Equivalent (A V E) by March 1, 2025. Result: More than $8 million in A V E.

3. SHARED MEDIA STRATEGY

* Objective: Participate in at least 10 community events by March 1, 2025. Result: 20 community events.

* Objective: Schedule experts to provide remarks during at least five speaking engagements by March 1, 2025. Result: Nine expert speaking events (rotary clubs, chambers of commerce, and corporate retreats).

* Objective: Distribute 10,000 promotional items in various community events by March 1, 2025. Result: 10,813 promotional items distributed at community events.

4. OWNED MEDIA STRATEGY

* Objective: Publish at least three news stories to the university news website by March 1, 2025. Result: 3 news stories with combined 1,563 impressions.

* Objective: Achieve a 2% conversion rate on call-to-action in email marketing to health care organizations and educational institutions by March 1, 2025. Result: 5% call-to-action conversion rate.

* Objective: Achieve 10,000 views through partnership with the university's institutional social media accounts by March 1, 2025. Result: More than 23,000 views of institution-owned social media posts related to Q P R.

A note at the bottom states all objectives and results are for the campaign evaluation period from September 1, 2024, to March 1, 2025, unless noted.

Navigate back to Image 1:.

